# Multiyear phytoremediation and dynamic of foliar metal(loid)s concentration during application of *Miscanthus* × *giganteus* Greef et Deu to polluted soil from Bakar, Croatia

**DOI:** 10.1007/s11356-020-09344-5

**Published:** 2020-06-02

**Authors:** Valentina Pidlisnyuk, Pavlo Shapoval, Željka Zgorelec, Tatyana Stefanovska, Oleksandr Zhukov

**Affiliations:** 1 Department of Environmental Chemistry and Technology, Jan Evangelista Purkyně University in Ústí nad Labem, Králova výšina 3132/7, Ústí nad Labem, Czech Republic; 2 Department of Physical, Analytical and General Chemistry, National University “Lvivska Polytechnika”, Sv.Yura Square 9, Lviv, 79013 Ukraine; 3 Department of General Agronomy, University of Zagreb, Svetošimunska cesta 25, 10000 Zagreb, Croatia; 4 Department of Plant Protection, National University of Life and the Environmental Sciences, Gerojiv Oboronu 13, Kyiv, Ukraine; 5 Bogdan Khmelnitsky Melitopol State Pedagogical University, Hetmanska St., 20, Melitopol, 72318 Ukraine

**Keywords:** Metal(loid)s polluted soil, Rijeka-Bakar area, *Miscanthus × giganteus*, Phytoremediation, Dynamic of foliar concentration

## Abstract

The multiyear cultivation of *Miscanthus* × *giganteus* Greef et Deu (*M.*×*giganteus*) at the soils polluted by metal(loid)s were researched. The biomass parameters and concentrations of elements: Ti, Mn, Fe, Cu, Zn, As, Sr, and Mo were determined in the plant’s organs at harvest. The same metal(loid)s were monitored in the plant’s leaves throughout three vegetation seasons. The principal component analysis and general linear model approaches were applied for statistical evaluation followed by Box-Cox transformation. The difference in the distribution of elements in the plant, the content of elements in the soil, various regime of uptake to the plant tissues, and the year of vegetation were analyzed as driving factors of the phytoremediation. The results showed that the leading promoter was the factor of the zone, which was the most essential for Ti, Fe, and Cu and the smallest for Mn. The factor of differences in soil pollution was essential for Zn and Mo, much less for As, Sr, and Mn, limited for Fe, and was not seen for Ti and Cu. The factor of the interrelation effects of the zone and experiment reflected the different regime of uptake for the plant tissues was seen for two elements: more prominent for Cu and smaller for Ti. While analyzing the dynamic of foliar concentrations of the metal(loid)s during 3 years, two groups were defined. Firstly, Fe, Ni, Mn, and Sr showed stable curves with limited distribution of the plant life cycle. Secondly, As, Zn, Cu, and Mo showed different fluctuations in the curves, which can be attributed to essential influence of those elements to the plant life cycle. Further research will be focused on the application of *M.*×*giganteus* to the polluted soil in a bigger scale and comparison results of laboratory and field experiments.

**Electronic supplementary material:**

The online version of this article (10.1007/s11356-020-09344-5) contains supplementary material, which is available to authorized users.

## Introduction

**Fig. 1:**
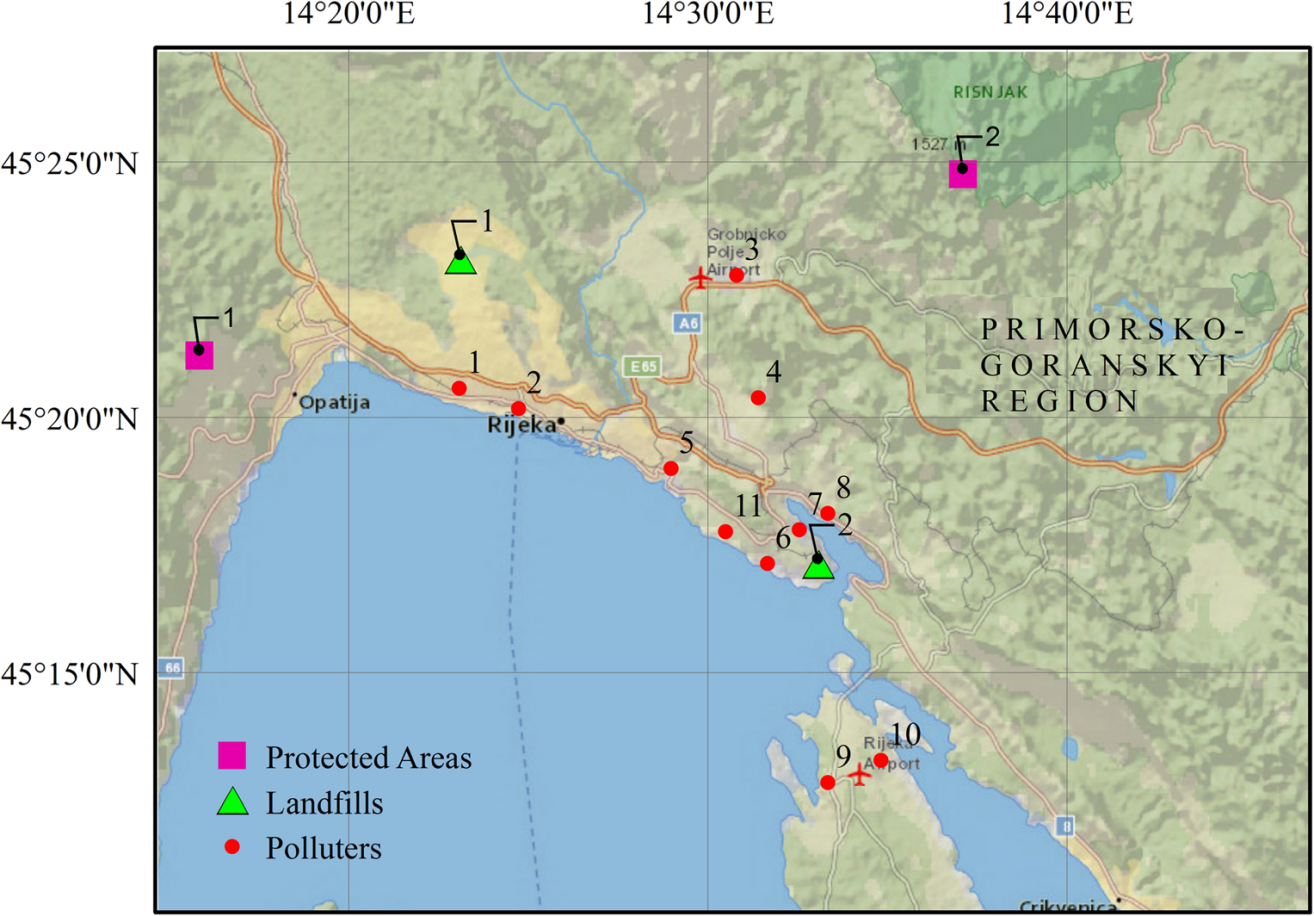
The map of point source polluters in Rijeka-Bakar area, Croatia (source: Google Maps—edited by Iva Hrelja, 2017; Hrelja et al. 2020). Polluters: 1. Shipyard “3. Maj”; 2. Oil refinery INA d.d.—Mlaka plant (closed); 3. Racetracks and airport Grobnik; 4. Industry Zone d.o.o. Bakar; 5. Shipyard “Viktor Lenac”; 6. Oil Refinery INA d.d.—Urinj Plant; 7. Coke Plant Bakar (closed); 8. Bulk Cargo Terminal; 9. DINA d.d.—Organic Petrochemical Company; 10. Rijeka Airport; 11. Power Plant Rijeka. Landfills: 1. Jama Sovjak; 2. Šoići. Protected areas: 1. Nature Park Učka; 2. National Park Risnjak

Industrial activities represent a valuable source of polluting the environment by toxic elements, which can be introduced into the atmospheric, aquatic, and terrestrial ecosystems (Vareda et al. 2019; Naila et al. 2019; Hui 2020; Rolka et al. 2020). In particular, the negative effect is exacerbated when an industrial zone is located near the water body, and pollutions can move directly to aquifer with additional contamination of the water biota as it was reported for the Venice Lagoon (Gieskes et al. 2015). Another negative case is presented by an industrially polluted zone of the port of Rijeka and Bakar Bay, Croatia where industrial activities between 1970 and 1990 caused regional pollution (Alebic-Juretic 1994; Jakšić et al. 2005). The locations of the main industrial entities in Bakar Bay region are presented in Fig. 1. Since the mid-1990s and after the war, most industries such as the coke factory were cut down, however, the area of Rijeka-Bakar is still considered as highly polluted area (Popadić et al. 2013) with tremendous negative impact to the Northern Croatian Adriatic coast including deterioration of water, sediments, soil (Jakšić et al. 2005; Bihari et al. 2007; Alebić-Juretić 2011; Cukrov et al. 2014; Hrelja et al. 2020), bay biodiversity (Ozretić et al. 1990; Perić et al. 2012), and human health (Bartoniček-Brgić and Matković 1989). The urgent request exists to develop the technology for remediation of the area, which has to be effective, not costly, and ecologically friendly.

Varieties of in situ and ex situ approaches are used for remediation of such areas, including surface capping, encapsulation, landfilling, soil flushing or washing, stabilization, solidification and phytoremediation and bioremediation (Liu et al. 2018; Burges et al. 2018). While the main techniques are rather costly and environmentally disturbing, the application of phytoremediation sounds promising, in particular with plants produced a high amount of biomass (Yadav et al. 2018; Antonkiewicz et al. 2019), and was recommended for post-industrial sites (Cunningham and Ow 1996; Chaney et al. 2014). Guarino and Sciarrilli (2017) reported successful application of phytoremediation using two plants with high annual biomass yield: *Helianthus annuus* L. and *Brassica juncea* L. when a considerable amount of toxic elements (Cd, Hg, Zn) were taken from soils of the industrial area of Porto Marghera. Phytotechnology with another high biomass yield crop *M.*×*giganteus* was proposed for revitalizing of post-mining, technosols, and smelting sites (Wanat et al. 2013; Chaney et al. 2014; Nsanganwimana et al. 2016). Recently, this plant was applied (Kharitonov et al. 2019) for phytoremediation of the industrial zone in Ukraine to soil contended large concentrations of metal(loid)s, industrial disposal in Serbia (Drazic et al. 2017), and post-industrial heavy polluted by metalloids post-mining sites in Poland (Rusinowski et al. 2019). However, there are not many researches on long-term application of *M.*×*giganteus* to post-industrial soil polluted by different metal(loid)s, and only a few publications (Nsangawimana et al. 2015; Pidlisnyuk et al. 2019) reported data on foliar metal(loid)s concentration during vegetation, which may give insight to the process.

The goals of the current study was to test the application of *M.*×*giganteus* as phytoremediation agent to the heavy polluted by metal(loid)s post-industrial soil from Bakar, Croatia, to measure the phytoremediation parameters during three vegetation seasons and to observe the peculiarities of metal(loid)s behavior in the plants’ organs in the long term. It was intended to define the main promoter factor of the process among differences in element concentration in the soil, years of vegetation, and elements’ nature and to compare the behavior of the metal(loid)s in plant’s foliar during multiyear growing.

## Materials and methods

### The origin of soils

The polluted soil was taken at Bakar industrial zone, Croatia, from the site with the following coordinates: 45° 17′ 56.7″ N 14° 32′ 34.4″ E; the soil was sampled on 13 April 2015. The non-polluted soil was taken from the agricultural field located in the village of St. Helena, Zagreb region, with the coordinates: 45° 90′ 50.60″ N and 16° 25′ 79.89″ E; the non-polluted soil was sampled on 14 April 2015. The soil sampling was done by the ISO standard (ISO 11464, 2001). The process was described in detail at Pidlisnyuk et al. (2018). By classification, the polluted soil was Technosols, and non-polluted soil was Stagnosols (FAO 2015).

### Experiment design

The pots used in the experiment had a volume of 20 l; in each pot, 1 kg of drainage was put to the bottom, followed by 14 kg of the research soil. The non-polluted soil was diluted by polluted soil in the following proportions:

**Table:** 

Scheme of the experiment	1	2	3	4	5
Weight of the non-polluted soil (100% )	100	75	50	25	–
Weight of the polluted soil (100% )	–	25	50	75	100

Receiving mixture soils were used for the experiment, which had two replications marked as A and B. The agrochemical parameters of soils were analyzed before the experiment start using the standard’s approaches. Data are presented in Table 1.

**Table 1:** The agrochemical parameters of the polluted and non-polluted soils (average ± standard error)

Soil parameter	Polluted soil	Non-polluted soil	Standard
рН (water extracted)	8.6 ± 0.60	7.5 ± 0.51	DSTU 8346:2015
рН (salt extracted)	7.7 ± 0.54	6.4 ± 0.40	GOST 26483-85
Total salinity (mg/100 g)	89 ± 5.56	80 ± 5.60	DSTU 7827:2015
Specific conductivity (μS/cm)	0.2 ± 0.01	0.2 ± 0.01	DSTU 8346:2015
Sum of adsorbed alkaline (mmol-equivalent/100 g)	1.0 ± 0.05	8.0 ± 0.52	GOST 27821-88
Available S (mg/kg)	1.6 ± 0.10	5.3 ± 0.31	DSTU 8347:2015
Nitrate N (mg/g)	13.2 ± 0.76	7.6 ± 0.48	DSTU 7629:2014
Alkaline hydrolyzed N (mg/kg)	5.2 ± 3.35	137 ± 7.90	DSTU 7863:2015
Available P (mg/kg)	47 ± 3.08	238 ± 16.27	DSTU 4115:2002
Available K (mg/kg)	222 ± 13.40	404 ± 27.70	DSTU 4115:2002
Available Ca (mmol-equivalent/100 g)	5.8 ± 0.32	10.0 ± 0.51	GOST 26487-85
Available Mg (mmol-equivalent/100 g)	1.5 ± 0.08	1.9 ± 0.10	GOST 26487-85
Organic matter (%)	6.7 ± 0.48	8.2 ± 0.59	DSTU 7632:2014

It may be concluded that non-polluted soil had an excellent quality being rich in the content of primary nutrients: K, P, and organic matters; however, the content of N was not entirely sufficient; that soil also had both measured pH (water and soil extracted) in neutral diapason, while polluted soil was light alkaline. The polluted soil was pure in the content of P and alkaline hydrolyzed N.

One rhizome of *M.*×*giganteus* was planted in each of the pots with an average weight of 20 g. Rhizomes were 3 years old and purchased at the agricultural station “Butsha” (Slovakia). The pots with plants were stored at the open-air throughout vegetation season and watered while necessary by pot water. Upon the end of the vegetation, the above part biomass was harvested and weighted. After that, pots with soil and roots were stored in a dark room throughout winter without watering. The pots were then exposed to light as soon as the first green shoots of *M.*×*giganteus* appeared. The experiment continued for three vegetation seasons in 2015–2016–2017, started on April 15, 2015, when rhizomes were planted to the pots and finished on December 12, 2017. At the end of each vegetation, the samples of plant’s organs, leaves, stems, and roots were collected randomly and analyzed for the content of metal(loid)s. The preparation of the plant’s organs for the analysis and the analysis breakdown was described in detail earlier (Pidlisnyuk et al. 2018). In parallel, the monitoring of elements’ content in the leaves of the plant was done throughout three vegetation seasons (2015–2016–2017). For that, the randomly selected sample of leaves was occasionally taken during vegetation and analyzed.

### Analysis of elements in the soil and plant organs

The content of elements in the soil and plant’s organs was analyzed using X-ray fluorescence analysis. The following metal(loid)s were under the investigation: Ti, Mn, Fe, Cu, Zn, As, Sr, and Mo; other elements were neglected being detected in minimal concentrations. Two types of soils were taken for analysis: one sample was taken from the surface of the pot, and another sample was taken from inside of the pot near the roots. The preparation of soil samples and analysis breakdown was described in detail at Pidlisnyuk et al. (2018). Data about elements’ content in the research soils are presented in Table 2.

**Table 2:** The concentrations of metal(loid)s (mg/kg dry wt.) in the mixtures of contaminated and clean soils (average ± std. error, *n* = 2) and Spearman rank-order correlations with contamination level (only statistically significant coefficients with *p* < 0.05 are shown)

Experiment			Zone	Ti	Mn	Fe	Cu	Zn	As	Sr	Mo
Clean	Contaminated	Contamination level									
			Limit value¤		750		100	200	30	100	
100%	0%	1	Surface	10,227 ± 738	665 ± 4	46,730 ± 5452	72 ± 7.5	265 ± 6	14.5 ± 2.5	127 ± 25.5	-
			Near roots	9206 ± 494	725 ± 121	50,997 ± 9367	77 ± 3.5	352 ± 59	11.0 ± 1.5	143 ± 41.0	-
75%	25%	2	Surface	7159 ± 279	750 ± 5	47,301 ± 4355	132 ± 11.5	765 ± 117	18.0 ± 4.0	117 ± 11.5	21.5 ± 0.5
			Near roots	6896 ± 72	747 ± 104	54,443 ± 6626	107 ± 6.5	531 ± 114	14.0 ± 1.0	118 ± 13.5	19.5 ± 3.5
50%	50%	3	Surface	4693 ± 633	803 ± 3	48,704 ± 2150	177 ± 21.0	1072 ± 78	19.0 ± 5.0	108 ± 9.5	50.0 ± 7.0
			Near roots	4961 ± 154	818 ± 45	56,651 ± 7614	152 ± 5.0	945 ± 186	16.0 ± 2.0	101 ± 0.5	46.5 ± 4.5
25%	75%	4	Surface	2882 ± 276	806 ± 25	55,626 ± 8152	209 ± 18.5	1347 ± 100	24.0 ± 2.0	105 ± 5.0	67.5 ± 0.5
			Near roots	3669 ± 43	858 ± 55	57,017 ± 10,408	224 ± 4.0	1496 ± 105	29.0 ± 3.0	100 ± 4.0	53.0 ± 7.0
0%	100%	5	Surface	2151 ± 179	844 ± 28	54,596 ± 6087	265 ± 12.0	1530 ± 50	27.0 ± 2.0	87 ± 4.5	86.0 ± 6.0
			Near roots	2981 ± 305	898 ± 31	60,408 ± 8288	266 ± 5.5	1840 ± 51	31.5 ± 4.5	113 ± 6.5	64.5 ± 14.5
Spearman correlation			Surface	− 0.98	0.91	–	0.96	0.98	0.83	− 0.69	0.99
			Near roots	− 0.98	0.71	–	0.98	0.98	0.94	–	0.89

¤Limit values of trace elements in the soil as for Croatia (European Commission 2018)

As expected, the element concentrations were increased as a proportion of the polluted soil increase in the mixture. However, those differences varied depending on the elements. The highest increase was observed for Cu, Zn, and Mo. The last element was not detected in the non-polluted soil. The concentrations of Fe and Sr were close. Surprisingly, the concentration of Ti was higher in the non-polluted soil than in the polluted soil, and this might be due to the local background.

### Statistics

The principal component analysis (PCI) and general linear models (GLMs) were applied for statistical evaluation using Box-Cox transformation. Тhe GLMs is an approach used as a numerical solution of ordinary differential equations (Butcher 2016). GLMs were used to assess the effect on the concentration of heavy metals in plant tissues through the categorical predictors (qualitative predictors) − Zone (the effect of the plant zone—roots, leaves, stems), Experiment (the effect of the experiment treatments), and continuum predictors (numerical predictors) − Day (day duration of the vegetation in time of the data collection), as well as their interactions (Zone × Experiment, Zone × Day, Experiment × Day). The calculations were performed in the program Statistica 12.0 (StatSoft Inc., Data Analysis Software System).

The normality is an essential assumption for many statistical techniques, and if data is not normally distributed, applying a Box-Cox makes it possible to run parametric statistical methods. A Box-Cox transformation was applied for the evaluation of research data (Dag et al. 2014). That approach is a way to transform non-normal dependent variables into a normal shape. The Box-Cox transformation has the form:

$$ y\left(\lambda \right)=\left\{\begin{array}{c}\frac{y^{\lambda }-1}{\lambda },\mathrm{if}\ \lambda \ne 0\\ {}\log y,\mathrm{if}\ \lambda =0\end{array}\right.. $$

where *y* is the data to be transformed and *λ* is the transformation exponent.

At the core of the Box-Cox transformation is an exponent, lambda (*λ*), which varies from − 5 to 5 (Asar et al. 2017). By considering all values, the optimal value that resulted in the best approximation of a normal distribution curve for data was selected. The procedure of the optimal lambda value searching was done utilizing the library AID (Dag and Ilk 2017) for a Language and Environment for Statistical Computing R (R Core Team 2018). A Spearman rank-order correlation coefficient was applied to reveal the correlation between metal(loid)s concentration and the pollution levels. The GLMs were used to test the significance of the effect of the plant zone (roots, leaves, stems), experimental treatments, and duration of the vegetation on the concentrations of the elements. The PCA was used for dimensionality reduction (Jolliffe 2002). It was applied based on the correlation matrix (as a concentration of elements is on a different scale) and presented in the form of so-called distance biplot.

## Results and discussion

The weight of the harvested dry biomass is presented in Table 3 and showed that the amount was almost the same during three vegetations. As reported (Lewandowski et al. 2000), the development of *M.*×*giganteus* improved with vegetation, and plantation intends to be almost established after three vegetation seasons producing a stable amount of biomass with a much higher harvest than in the first 2 years of establishing. That did not happen in the current laboratory experiment, which could be explained by limited surface for root’s development in the pots. As expected, the highest value of biomass was received when *M.*×*giganteus* grew in the non-polluted soil (1A and 1B). Moreover, with a dilution of the non-polluted soil even by 25% of polluted Bakar soil, the value of harvested biomass substantially decreased (2A and 2B). The further soil dilution impacted the decreasing only slightly (3A and 3B) and was almost the same when biomass produced at only polluted soil (5A and 5B).

**Table 3:** Dry *M.*×*giganteus* biomass (leaves and stems), g/per treatment at three harvests

Year	Harvested day	Seria	Pollution level				
			1	2	3	4	5
2015	November 21	A	132.77	80.36	55.65	48.65	50.76
		B	147.23	76.92	52.32	45.93	42.62
2016	December 22	A	129.95	78.65	47.81	54.85	45.58
		B	148.45	69.92	50.60	44.77	42.87
2017	December 16	A	125.51	74.09	40.82	57.84	41.62
		B	145.02	67.59	46.75	41.06	39.59

The concentration of metal(loid)s (mg/kg dry weight) in plant organs upon harvest is presented in Table 4. For that calculation, all replicates measured for one element were joined together.

**Table 4:** The concentration of elements (mg/kg dry weight) in the plant organs of *M.*×*giganteus* (average ± std. error)

Plant organs	Year	N*	Ti	Mn	Fe	Cu	Zn	As	Sr	Mo
Roots	2015	10	714 ± 102	32 ± 1.9	5373 ± 376	34 ± 5.3	178 ± 28	1.24 ± 0.25	28.7 ± 2.7	5.7 ± 0.9
	2016	10	799 ± 103	166 ± 8.3	6621 ± 211	48 ± 11.4	165 ± 35	3.05 ± 0.28	17.4 ± 1.8	4.8 ± 1.0
	2017	10	689 ± 116	115 ± 11.1	6251 ± 529	63 ± 10.7	244 ± 42	4.44 ± 0.64	43.9 ± 4.4	13.8 ± 2.3
Leaves	2015	20	209 ± 46	18 ± 1.4	119 ± 6.1	4.1 ± 1.2	45 ± 5.6	2.41 ± 0.56	12.5 ± 0.6	2.3 ± 0.2
	2016	60	109 ± 6.3	101 ± 14.8	216 ± 13	8.0 ± 1.3	72 ± 5.7	1.40 ± 0.09	14.9 ± 1.4	4.3 ± 0.3
	2017	30	47 ± 2.8	48 ± 3.4	219 ± 28	9.5 ± 1.6	46 ± 4.0	1.13 ± 0.12	10.6 ± 0.9	4.8 ± 0.5
Stems	2015	10	33 ± 2.9	21 ± 1.8	126 ± 2.6	2.0 ± 1.0	203 ± 35	0.27 ± 0.03	7.7 ± 0.7	3.9 ± 0.5
	2016	10	24 ± 4.0	38 ± 7.4	41 ± 6.9	5.9 ± 0.7	312 ± 40	0.76 ± 0.10	9.6 ± 1.1	2.4 ± 0.4
	2017	10	14 ± 1.2	30 ± 4.1	42 ± 4.9	2.4 ± 1.2	182 ± 35	0.20 ± 0.02	2.7 ± 0.2	0.5 ± 0.1

*The number of the replicates

**Fig. 2:**
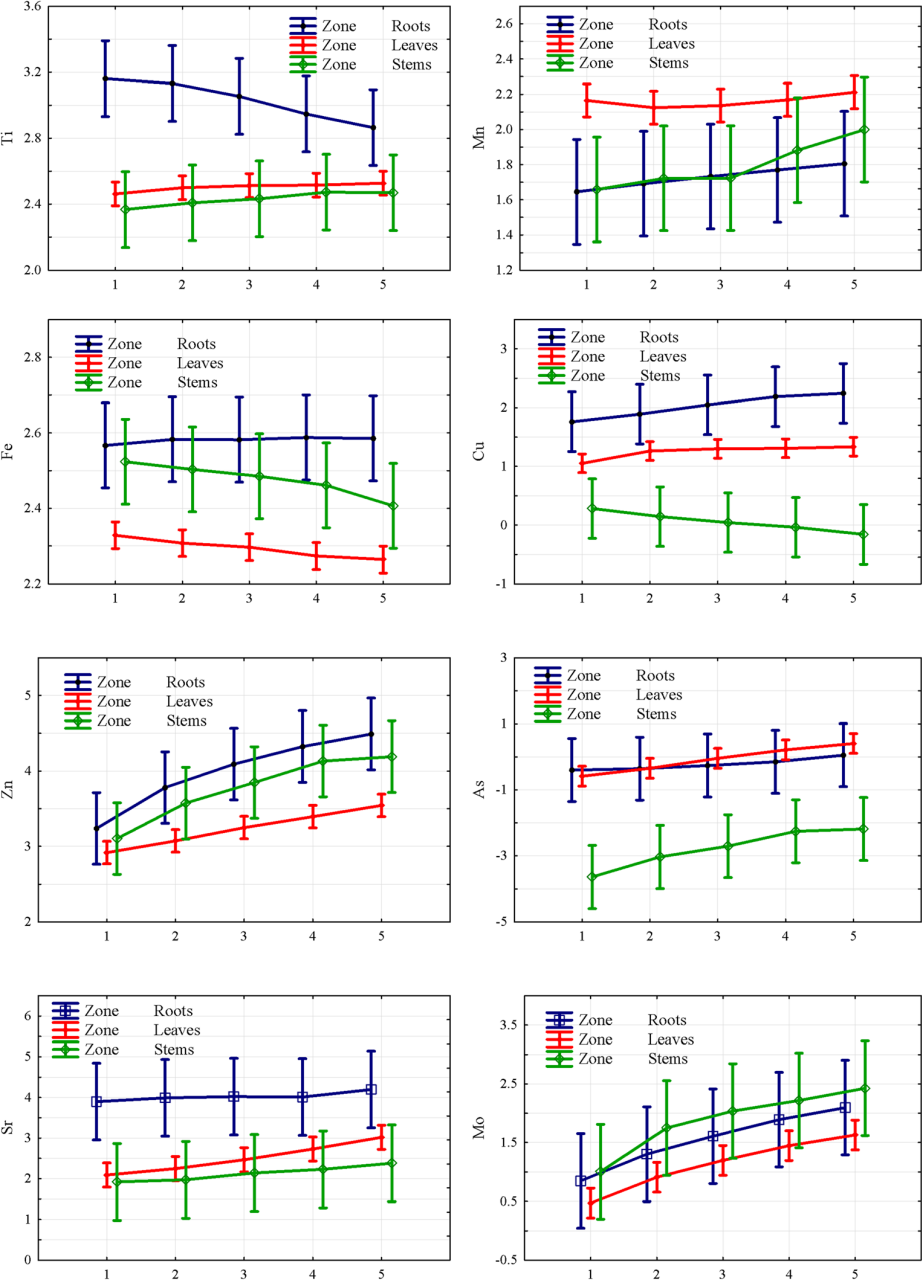
The concentration of the elements in *M.*×*giganteus* organs (variable “Zone”) depending on the level of soil pollution (time is presented as covariant) using the GLMs model. In this model, the abscissa axis is soil contamination level presented as the attracted level of soil dilution; the ordinate axis is attracted concentrations of elements (mg/kg) after the Box-Cox transformation (average ± standard deviation)

In Table S1–8 (supplemented materials), the statistically evaluated results on the dependence of each monitored element concentrations in the different plant’s organs as impacted by the level of soil pollution by that element are presented. The impact of the metal(loid)s’ nature to the distribution between the plant’s parts depending on the level of soil pollution was summed up in Fig. 2.

It can be concluded that the phytoremediation behavior of elements in plant tissues was different and could be grouped as follows: for Zn and Fe, the situation was the same as for *M.*×*giganteus* phytoremediation when plants grew in slightly metal(loid)s polluted post-military soil (Pidlisnyuk et al. 2019). For the case of heavy polluted post-mining soils (Rusinowski et al. 2019), the highest concentration of elements was distributed to the roots, followed by stems and leaves. This distribution is typical for the phytostabilization process observed with *M.*×*giganteus* when the crop is applied to the metal(loid)s contaminated soils (Nsanganwimana et al. 2014). For Ti, Cu, and Sr, the highest amount also was observed in the roots, however, followed by leaves and then by stems. The behavior of Mn and Mo was different from the highest distribution to above part biomass (leaves in the case of Mn and stems in the case of Mo) and less distribution was to the roots.

When analyzing the response of different parts of the plant to increasing concentration of the elements in the soil, it may be concluded that in the case of Zn, As, and Mo, the increase led to direct increase of concentration in all vegetation parts. For Ti, the roots showed a decrease of the uptake with an increasing concentration, whereas the leaves and stems almost did not react to an increase of concentration. For Mn, the plant almost did not exhibit any reaction in all its parts with the increasing of concentration. For Fe, the roots demonstrated no response for an increasing concentration, whereas a decrease of uptake in the above part of the plant was measured with an increase of concentration in the soil. For Cu, an increase of the element in the soil yields an increase in the roots, whereas the leaves showed no change, and the stems exhibited a decrease. For Sr, the roots showed no changes, whereas a slight increase was observed for the leaves and the stems.

**Fig. 3:**
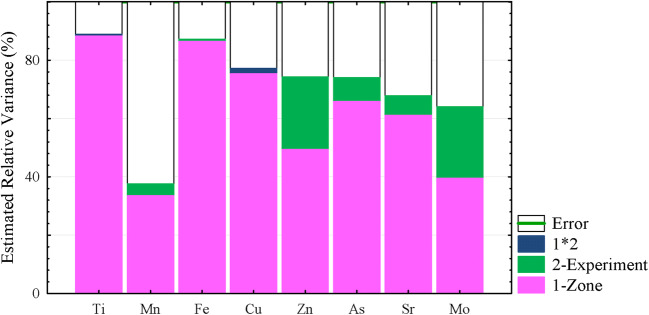
The components of the element concentration variation (after Box-Cox transformation) depended on plant organs (variable 1—“Zone”), experiment treatment (variable 2—“Experiment”), and its interaction (1*2) (with vegetation duration as a covariate). Zone—the effect of the plant organs (roots, leaves, stems); Experiment—the effect of the experiment treatments (levels 1–5); 1*2—the interaction effects of the Zone and Experiment.

For an understanding of metal(loid)s’ behavior and defining the impact of each factor, the components of the concentration of the element vary depending on the experiment treatment (dilution of the soil) and plant zone (roots, leaves, stems) was analyzed. The results are illustrated in Fig. 3.

It can be seen that the primary source of the metal(loid)s concentration variation was a factor of plant organs (variable “Zone”), i.e., redistribution between parts of the plant, which was the most essential for Ti, Fe, and Cu and the smallest for Mn. That factor was stressed as important earlier (Medina et al. 2003; Kabata-Pendias 2010). The factor experiment (different levels of soil contamination—variable “Experiment”) was the most essential for Zn and Mo, however, much less for As, Sr, and Mn, limited for Fe, and was not observed for Ti and Cu. The factor of the interaction effects of the Zone and Experiment (1*2), which reflected the different regimes of uptake for the plant organs was observed for two elements only: bigger for Cu and smaller for Ti, and for other elements that factor was neglected.

**Fig. 4:**
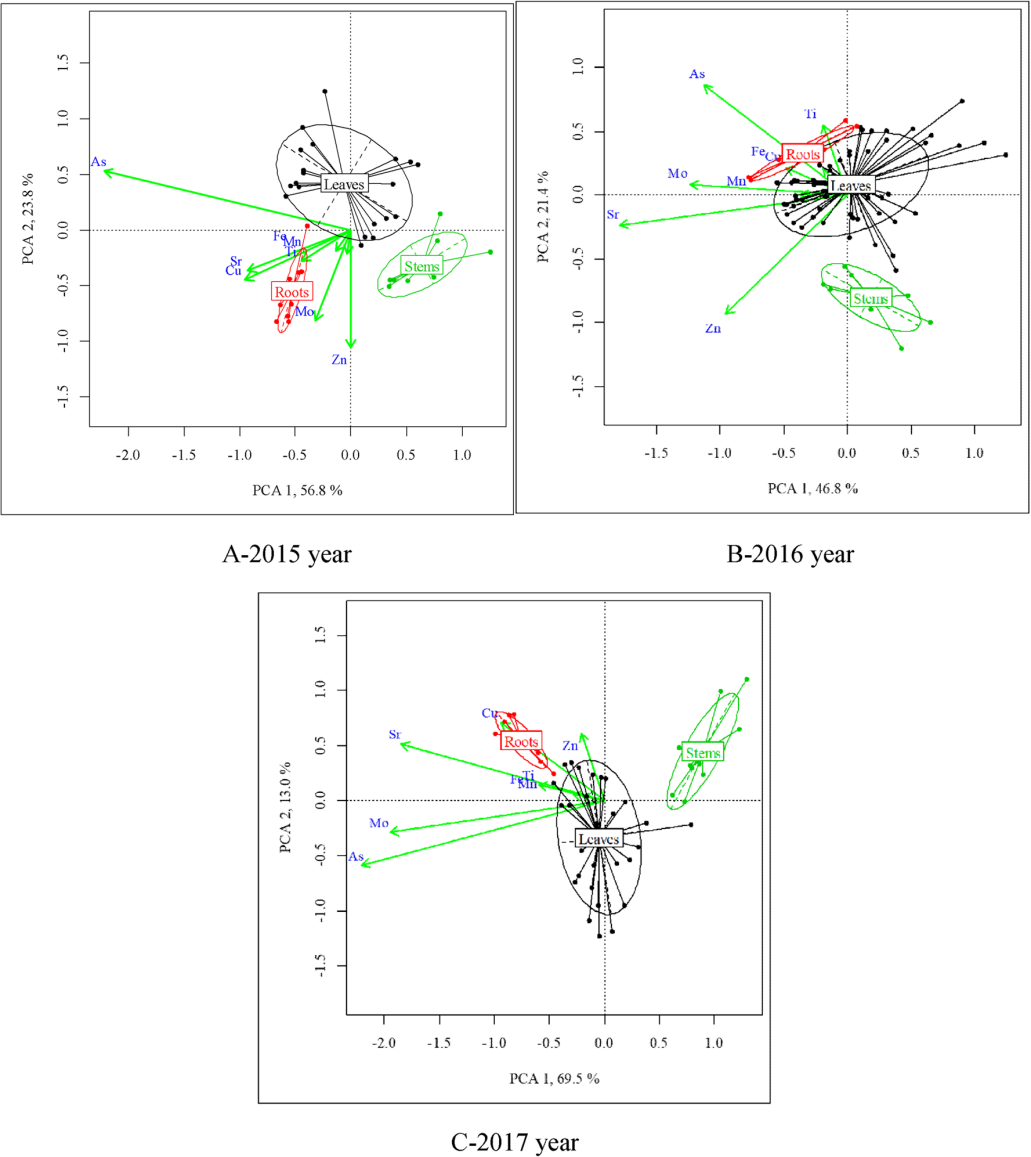
(A)–(C) The application of PCA for to the research system when *M.*×*giganteus* was grown during three vegetation seasons in the metal(loid)s polluted soil

The PCA was used for the generalized samples for 3 years and permitted to determine the specific principal components for each year, which eigenvalues exceed 1. By this criterion, principal components 1 and 2 were selected to be considered further. The principal component 1 in the annual samples explained 46.8–69.5% of the total concentration variability of the studied elements. To the greatest extent, this component reflected the variety of all metal(loid)s concentration except for Zn in 2015, and these elements are characterized by the same sign of correlation coefficient. Thus, principal component 1 can be meaningfully considered the total level of metal(loid)s and distinguishes the sensitivity of different morphological parts of the plant to the degree of soil pollution. This assumption is confirmed by the general linear model: the effect of the “Experiment” factor is statistically significant (Table S9—supplemented materials). It should also be noted that there is no statistically significant interaction between the morphological parts of the plant (“Zone”) and the pollution level (“Experiment”) in its effect on the principal component 1. That is why the principal component 1 may be concluded to reflect the synchronous changes in the physiological process of metal(loid)s uptake within plant organs induced by the total exogenous cause. The soil pollution level is such an exogenous cause. This result may be illustrated by Fig. 4, which shows that increasing of the total level of pollution increases proportionally the content of metal(loid)s in the plant organs while the ratio of its concentration remains unchanged.

Metal(loid)s were accumulated in roots to the greatest extent, followed by leaves and stems. The vegetation duration significantly impacted the accumulation (uptake) of elements by plant organs (significance level 0.067). As a result, this very well expresses that trend is orientated toward decreasing the content of metal(loid)s. It can be assumed that mechanisms counteract the entry of metal(loid)s into the plant developed during its growth.

The principal component 2 explained 14.7% of the total concentration variability of the studied elements in the generalized sample or 13.0–23.8% in annual samples. This component is most sensitive to the opposite trend in the dynamics of As and Zn concentrations. The analyses of the dynamics of the principal component based on the level of pollution indicated that at high levels of soil pollution, the concentration of Zn in the plants increased while the concentration of As decreased. The regression coefficient of the duration of the vegetation indicated that this trend increased with duration time (Table S10—supplemented materials). The ratio of Zn to As concentrations between the plant organs did not change with increasing pollution levels. Thus, principal component 2 can be considered as marker of the endogenous plant response to soil pollution by metal(loid)s. The PCA for each year showed an annual invariance of the relationship discussed earlier between the concentration of metal(loid)s in the plant depending on the level of pollution.

**Fig. 5:**
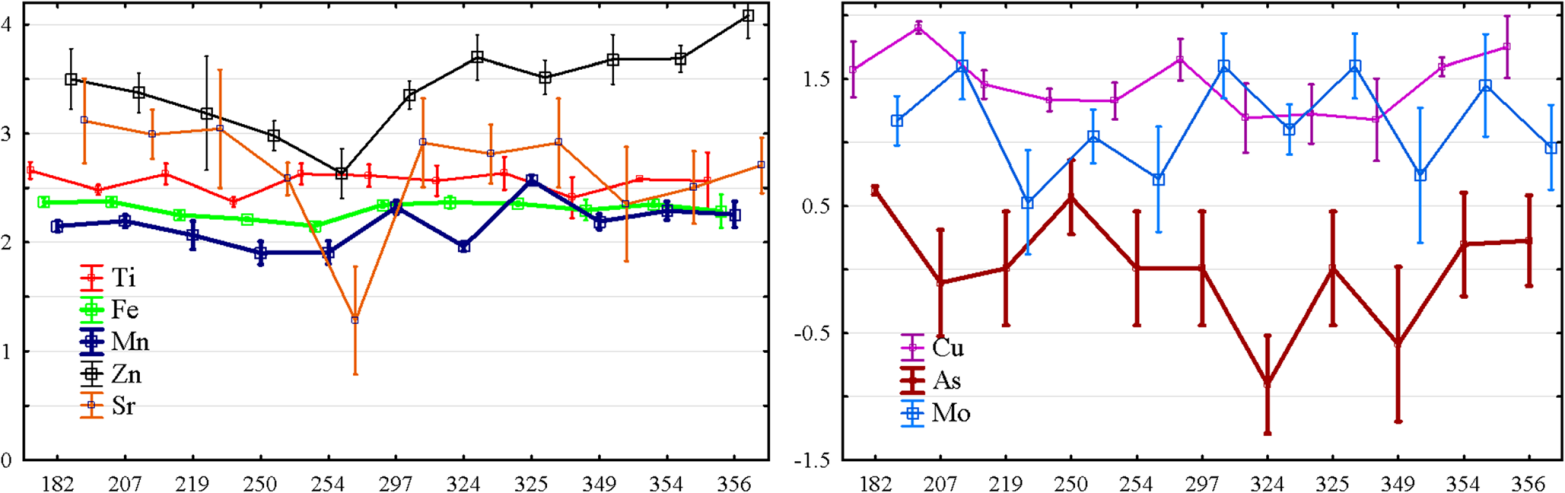
The dynamic of metal(loid)s concentration in the leaves of *M.*×*giganteus* during vegetation period: *x*-axes—the day of vegetation; *y*-axes—concentration of metal(loid)s (in mg/g); time zero indicates planting time

**Fig. 6:**
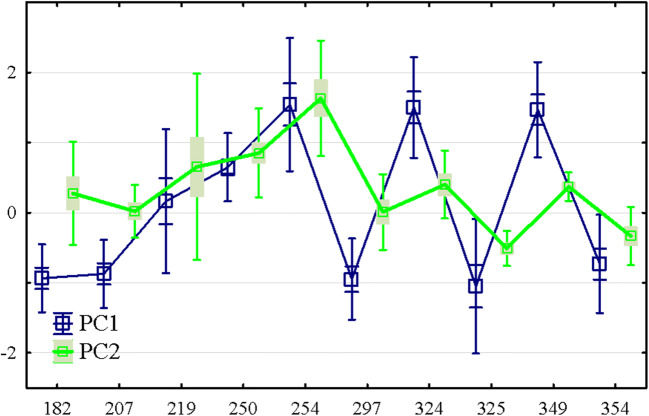
The dynamic of the principal components PC1 and PC2 with the time of vegetation: *x*-axes—the day of vegetation; *y*-axes—PC1 and PC2 scores; time zero indicates planting time

Figure 5 presents the dynamics of the concentration of individual metal(loid)s’ variability over time. These dynamics are interrelated, and the nature of this relationship was earlier discussed through the analysis of the principal components. It is also possible to display the weight of each variable for each change (principal component scores) in the temporal dynamics similar to the concentration of an individual metal(loid). However, for this case (Fig. 6), it is referred to the temporal dynamics of principal components 1 and 2.

The principal component 1 demonstrated the oscillation dynamics in time and described the overall level of metal(loid)s in the plant. It was suggested that the peaks and drops in the metal(loid) contents are associated with the stages of the plant life cycle. For Fe, Ni, and Mn, the curve was almost stable and not determined by the vegetation period, and the only unexpected decrease for Sr may be referred to as the error with significant probability. So, this group of elements mainly not disturbed the plant life cycle. For other elements, As, Zn, Cu, and Mo, the fluctuation was evident, so the existence of those elements in the soil impacted the plant’s life cycle differently. The principal component 2 indicated the different dynamics of the As and Zn contents. The analysis of the temporal dynamics of the principal component 2 showed a maximum concentration of As and a minimum concentration of Zn in the middle period of vegetation of *M.*×*giganteus*. It has to be emphasized that the principal component 2 described the minor fraction of metal(loid)s’ variability. Therefore, the established trend was not evident in the case of directly considering the temporal dynamics of metal(loid)s. In this way, t As concentration reached the local minimum in the second half of the growing season, while Zn showed a local minimum in the first half of the growing season. The uncertainty appeared perhaps because the roles of Zn and As were prominent in the formation of dynamics, which was described as the principal components 1 and 2, and were orthogonal (independent) by definition. In other words, at least two independent and essential processes were critical in the dynamics of these two metal(loid)s. Most likely, these are the physiologically normal difference in the distribution of metal(loid)s in the plant organs and the reaction of the plant to soil pollution by metal(loid)s.

The direct analysis of the temporal dynamics of Mo and Cu did not allow us to identify a marked trend of variation. The reason can be found in analyzing the principal components of varying the metal(loid)s concentration in the plant for each year separately. For most metal(loid)s, the influence of factors that manifested themselves in the principal components 1 and 2 remained invariant in time, while Mo and Cu represented an exception. Thus, PC2 played a crucial role in varying Mo concentration in 2015, whereas PC1 in 2016 and 2017. For Cu, PC1 played a crucial role in 2015, and consequently, during 2016 and 2017, the role of PC2 was strengthened while the influence of PC 1 remained. The additional research is needed to fully interpret the dynamics behavior of Mo and Cu because these two elements are sensitive to the varied factors of the different nature presented by the PC1 and PC2, and this sensitivity is changed considerably over time.

## Conclusion

The phytoremediation process with *M.*×*giganteus* in metal(loid)s polluted soil from Bakar, Croatia was studied 3 years in the laboratory conditions. Results showed the proper development of the plant at the highly polluted soil with sufficient biomass production. However, its volume decreased by increasing the level of soil pollution. The concentrations of monitored metal(loid)s: Ti, Mn, Fe, Cu, Zn, As, Sr, and Mo were measured continuously in the plant’s tissues at harvest, and through vegetation, the metal(loid) concentrations were monitored in *M.*×*giganteus* leaves as well. PCA and GLMs were applied for data evaluation, followed by using Box-Cox transformation. Results showed the differences of metal(loid)s’ behavior in the plants’ organs depending on vegetation year, the concentration of elements in the soil, and their nature. For Zn and Fe, the process was typical as for *M.*×*giganteus* phytostabilization: the highest concentration of those elements was detected in the roots, followed by stems and leaves. For Ti, Cu, and Sr, the highest uptake was observed to the roots, followed by the leaves and stems. For As, the uptake to the roots and leaves were almost the same while less metal(loid)s moved to the stems. For Mn and Mo, the highest uptake was observed in the upper part of the plant (leaves for Mn and stems for Mo), while fewer uptake was observed to the roots. The main reason for metal(loid) concentrations variation was a factor of the zone: redistribution between parts of the plant was the most essential for Ti, Fe, and Cu and the smallest for Mn. The factor experiment (different levels of soil pollution) was valuable for Zn and Mo, however, much less for As, Sr and Mn, limited for Fe, and was not observed for Ti and Cu. The factor of the interrelation effects of Zone and Experiment (1*2) reflected the different regimes of uptake to the plant tissues, which was observed for two elements: more prominent for Cu and smaller for Ti. For other elements, this factor was neglected.

The dynamic of foliar concentrations showed two main groups of the metal(loid)s depending on their influence on the stages of the plant’s life cycle. For Fe, Ni, Mn, and Sr, the dynamic curves were almost stable and not determined by the vegetation period, so this group of elements mainly not disturbed the plant’s life cycle. The second group of elements was presented by As, Zn, Co, and Mn, which migrations within the leaves varied differently during vegetation, so these elements had an essential influence on the stages of *M.*×*giganteus* life cycle.

Further research will be focused on *M.*×*giganteus* application to the post-industrial soil on a bigger scale and comparative correlative analysis between laboratory and field experiments peculiarities.

## Electronic supplementary material

ESM 1 (DOCX 36 kb).
