# Supplementary material for: Multiyear phytoremediation and dynamic of foliar metal(loid)s concentration during application of Miscanthus × giganteus Greef et Deu to polluted soil from Bakar, Croatia
Source: Environ Sci Pollut Res Int. 2020 Jun 2;27(25):31446–57. doi: 10.1007/s11356-020-09344-5 (PMC7651535; doi:10.1007/s11356-020-09344-5)
Supplement: Supplementary file 1 — (DOCX 36 kb). [file 11356_2020_9344_MOESM1_ESM.docx]

**Supplementary materials**

**Table S1**. General linear model of the Ti concentration (after Box-Cox transformation, *λ* = –0.27) dependence from the plant organs (variable “Zone”), experiment treatment, day of the vegetation, and their interrelation, *R_adj_^2^* = 0.77, *F*-ratio = 21.3, *p* < 0.001, Day regr. coef. –0.46±0.17

| Predictor | SS | Degr. of freedom | MS | *F*-ratio | *p*-value |
| --- | --- | --- | --- | --- | --- |
| Intercept | 2.62 | 1 | 2.62 | 105.3 | < 0.001 |
| Zone | 0.24 | 2 | 0.12 | 4.93 | 0.008 |
| Experiment | 0.21 | 4 | 0.054 | 2.16 | 0.076 |
| Day | 0.20 | 1 | 0.198 | 7.95 | 0.005 |
| Zone*Experiment | 0.42 | 8 | 0.052 | 2.09 | 0.040 |
| Zone*Day | 0.35 | 2 | 0.175 | 7.05 | 0.001 |
| Experiment*Day | 0.22 | 4 | 0.055 | 2.22 | 0.069 |
| Error | 3.68 | 148 | 0.025 | - | - |

Notes: Zone – the effect of the plant zone (roots, leaves, stems), experiment – the effect of the experiment treatments, Day – day duration of the vegetation in time of the data collection, Zone*Experiment, Zone*Day, Experiment*Day – the interrelation effects of the corresponding ordinal factors, SS – sum of squares, MS – mean of the sum of squares

**Table S2**. General linear model of the Mn concentration (after Box-Cox transformation, *λ* = –0.33) dependence from the plant organs (variable “Zone”), experiment treatment, day of the vegetation, and their interrelation, *R_adj_^2^* = 0.29, *F*-ratio = 4.31, *p* < 0.001, Day regr. coef. 1.43±0.30

| Predictor | SS | Degr. of | MS | *F*-ratio | *p*-valuel |
| --- | --- | --- | --- | --- | --- |
| Intercept | 0.001 | 1 | 0.001 | 0.03 | 0.87 |
| Zone | 0.78 | 2 | 0.39 | 9.32 | < 0.001 |
| Experiment | 0.11 | 4 | 0.028 | 0.68 | 0.61 |
| Day | 0.95 | 1 | 0.95 | 22.7 | < 0.001 |
| Zone*Experiment | 0.21 | 8 | 0.026 | 0.63 | 0.75 |
| Zone*Day | 0.78 | 2 | 0.39 | 9.35 | < 0.001 |
| Experiment*Day | 0.061 | 4 | 0.015 | 0.37 | 0.83 |
| Error | 6.17 | 148 | 0.042 | - | - |

Notes: Zone – the effect of the plant zone (roots, leaves, stems), experiment – the effect of the experiment treatments, Day – day duration of the vegetation in time of the data collection, Zone*Experiment, Zone*Day, Experiment*Day – the interrelation effects of the corresponding ordinal factors, SS – sum of squares, MS – MS – mean of the sum of squares

**Table S3**. General linear model of the Fe concentration (after Box-Cox transformation, *λ* = –0.37) dependence from the plant organs (variable “Zone”), experiment treatment, day of the vegetation, and their interrelation, *R_adj_^2^* = 0.82, *F*-ratio = 37.2, *p* < 0.001, Day regr. coef. –0.89±0.15

| Predictor | SS | Degr. of | MS | *F*-ratio | *p*-value |
| --- | --- | --- | --- | --- | --- |
| Intercept | 2.33 | 1 | 2.33 | 391.53 | < 0.001 |
| Zone | 0.40 | 2 | 0.20 | 33.24 | < 0.001 |
| Experiment | 0.003 | 4 | 0.001 | 0.15 | 0.96 |
| Day | 0.20 | 1 | 0.20 | 33.99 | < 0.001 |
| Zone*Experiment | 0.034 | 8 | 0.004 | 0.72 | 0.67 |
| Zone*Day | 0.46 | 2 | 0.23 | 38.30 | < 0.001 |
| Experiment*Day | 0.005 | 4 | 0.001 | 0.22 | 0.93 |
| Error | 0.88 | 148 | 0.01 | – | – |

Notes: Zone – the effect of the plant zone (roots, leaves, stems), experiment – the effect of the experiment treatments, Day – day duration of the vegetation in time of the data collection, Zone*Experiment, Zone*Day, Experiment*Day – the interrelation effects of the corresponding ordinal factors, SS – sum of squares, MS – MS – mean of the sum of squares

**Table S4**. General linear model of the Cu concentration (after Box-Cox transformation, *λ* = –0.30) dependence from the plant organs (variable “Zone”), experiment treatment, day of the vegetation, and their interrelation, *R_adj_^2^* = 0.63, *F*-ratio = 14.9, *p* < 0.001, Day regr. coef. 0.62±0.22

| Predictor | SS | Degr. of | MS | *F*-ratio | *p*-value |
| --- | --- | --- | --- | --- | --- |
| Intercept | 0.12 | 1 | 0.12 | 0.99 | 0.32 |
| Zone | 2.97 | 2 | 1.48 | 12.27 | < 0.001 |
| Experiment | 0.08 | 4 | 0.02 | 0.18 | 0.95 |
| Day | 0.98 | 1 | 0.98 | 8.11 | < 0.001 |
| Zone*Experiment | 1.85 | 8 | 0.23 | 1.91 | 0.06 |
| Zone*Day | 3.01 | 2 | 1.50 | 12.46 | < 0.001 |
| Experiment*Day | 0.10 | 4 | 0.03 | 0.21 | 0.93 |
| Error | 17.88 | 148 | 0.12 | – | – |

Notes: Zone – the effect of the plant zone (roots, leaves, stems), experiment – the effect of the experiment treatments, Day – day duration of the vegetation in time of the data collection, Zone*Experiment, Zone*Day, Experiment*Day – the interrelation effects of the corresponding ordinal factors, SS – sum of squares, MS – MS – mean of the sum of squares

**Table S5**. General linear model of the Zn concentration (after Box-Cox transformation, *λ* = –0.10) dependence from the plant organs (variable “Zone”), experiment treatment, day of the vegetation, and their interrelation, *R_adj_^2^* = 0.683, *F*-ratio = 18.5, *p* < 0.001, Day regr. coef. 0.23±0.20

| Predictor | SS | Degr. of | MS | *F*-ratio | *p*-value |
| --- | --- | --- | --- | --- | --- |
| Intercept | 1.89 | 1 | 1.89 | 17.73 | < 0.001 |
| Zone | 0.13 | 2 | 0.07 | 0.62 | 0.54 |
| Experiment | 2.07 | 4 | 0.52 | 4.87 | < 0.001 |
| Day | 0.14 | 1 | 0.14 | 1.32 | 0.25 |
| Zone*Experiment | 1.18 | 8 | 0.15 | 1.38 | 0.21 |
| Zone*Day | 0.21 | 2 | 0.10 | 0.97 | 0.38 |
| Experiment*Day | 0.74 | 4 | 0.18 | 1.74 | 0.14 |
| Error | 15.73 | 148 | 0.11 | – | – |

Notes: Zone – the effect of the plant zone (roots, leaves, stems), experiment – the effect of the experiment treatments, Day – day duration of the vegetation in time of the data collection, Zone*Experiment, Zone*Day, Experiment*Day – the interrelation effects of the corresponding ordinal factors, SS – sum of squares, MS – mean of the sum of squares

**Table S6**. General linear model of the As concentration (after Box-Cox transformation, *λ* = –0.36) dependence from the plant organs (variable “Zone”), experiment treatment, day of the vegetation, and their interrelation, *R_adj_^2^* = 0.62, *F*-ratio = 14.1, *p* < 0.001, Day regr. coef. 0.79±0.22

| Predictor | SS | Degr. of | MS | *F*-ratio | *p*-value |
| --- | --- | --- | --- | --- | --- |
| Intercept | 6.26 | 1 | 6.26 | 14.54 | < 0.001 |
| Zone | 9.53 | 2 | 4.77 | 11.07 | < 0.001 |
| Experiment | 0.04 | 4 | 0.01 | 0.02 | 1.00 |
| Day | 5.44 | 1 | 5.44 | 12.64 | < 0.001 |
| Zone*Experiment | 2.44 | 8 | 0.30 | 0.71 | 0.68 |
| Zone*Day | 8.86 | 2 | 4.43 | 10.29 | < 0.001 |
| Experiment*Day | 0.54 | 4 | 0.13 | 0.31 | 0.87 |
| Error | 63.7 | 148 | 0.43 | – | – |

Notes: Zone – the effect of the plant zone (roots, leaves, stems), experiment – the effect of the experiment treatments, Day – day duration of the vegetation in time of the data collection, Zone*Experiment, Zone*Day, Experiment*Day – the interrelation effects of the corresponding ordinal factors, SS – sum of squares, MS – mean of the sum of squares

**Table S7**. General linear model of the Sr concentration (after Box-Cox transformation, *λ* = 0.06) dependence from the plant organs (variable “Zone”), experiment treatment, day of the vegetation, and their interrelation, *R_adj_^2^* = 0.462, *F*-ratio = 7.7, *p* < 0.001, Day regr. coef. –0.39±0.27

| Predictor | SS | Degr. of | MS | *F*-ratio | *p*-value |
| --- | --- | --- | --- | --- | --- |
| Intercept | 4.79 | 1 | 4.79 | 11.42 | < 0.001 |
| Zone | 0.49 | 2 | 0.24 | 0.58 | 0.56 |
| Experiment | 0.09 | 4 | 0.02 | 0.05 | 0.99 |
| Day | 0.95 | 1 | 0.95 | 2.27 | 0.13 |
| Zone*Experiment | 1.21 | 8 | 0.15 | 0.36 | 0.94 |
| Zone*Day | 0.24 | 2 | 0.12 | 0.29 | 0.75 |
| Experiment*Day | 0.08 | 4 | 0.02 | 0.05 | 1.00 |
| Error | 62.08 | 148 | 0.42 | – | – |

Notes: Zone – the effect of the plant zone (roots, leaves, stems), experiment – the effect of the experiment treatments, Day – day duration of the vegetation in time of the data collection, Zone*Experiment, Zone*Day, Experiment*Day – the interrelation effects of the corresponding ordinal factors, SS – sum of squares, MS – mean of the sum of squares

**Table S8**. General linear model of the Mo concentration (after Box-Cox transformation, *λ* = –0.09) dependence from the plant organs (variable “Zone”), experiment treatment, day of the vegetation, and their interrelation, *R_adj_^2^* = 0.53, *F*-ratio = 10.2, *p* < 0.001, Day regr. coef. –0.74±0.24

| Predictor | SS | Degr. of | MS | *F*-ratio | *p*-value |
| --- | --- | --- | --- | --- | --- |
| Intercept | 4.67 | 1 | 4.67 | 15.25 | < 0.001 |
| Zone | 6.01 | 2 | 3.01 | 9.83 | < 0.001 |
| Experiment | 0.06 | 4 | 0.01 | 0.05 | 1.00 |
| Day | 2.79 | 1 | 2.79 | 9.13 | < 0.001 |
| Zone*Experiment | 0.27 | 8 | 0.03 | 0.11 | 1.00 |
| Zone*Day | 6.92 | 2 | 3.46 | 11.31 | < 0.001 |
| Experiment*Day | 0.44 | 4 | 0.11 | 0.36 | 0.84 |
| Error | 45.28 | 148 | 0.31 | – | – |

Notes: Zone – the effect of the plant zone (roots, leaves, stems), experiment – the effect of the experiment treatments, Day – day duration of the vegetation in time of the data collection, Zone*Experiment, Zone*Day, Experiment*Day – the interrelation effects of the corresponding ordinal factors, SS – sum of squares, MS – mean of the sum of squares

**Table S9.** General linear model of the PC1 dependence from the plant organs (variable “Zone”), experiment treatment (variable “Experiment”), day of the vegetation, and their interrelation, *R_adj_^2^* = 0.72, *F*-ratio = 30.3, *p* < 0.001, regression coefficient on the continual predictor “Day” (vegetation duration) is 0.09±0.05

| Predictor | SS | Degr. of | MS | *F*-ratio | *p*-value |
| --- | --- | --- | --- | --- | --- |
| Intercept | 5.0 | 1 | 5.0 | 4.0 | 0.046 |
| Zone | 504.4 | 2 | 252.2 | 205.1 | 0.000 |
| Experiment | 39.3 | 4 | 9.8 | 8.0 | 0.000 |
| Day | 4.2 | 1 | 4.2 | 3.4 | 0.067 |
| Zone*Experiment | 0.3 | 8 | 0.0 | 0.0 | 1.000 |
| Error | 189.4 | 154 | 1.2 | 0.0 | 0.0 |

Notes: Zone – the effect of the plant zone (roots, leaves, stems), experiment – the effect of the experiment treatments, Day – day duration of the vegetation in time of the data collection, Zone*Experiment, Zone*Day, Experiment*Day – the interrelation effects of the corresponding ordinal factors, SS – sum of squares, MS – mean of the sum of squares

**Table S10.** General linear model of the PC2 dependence from the the plant organs (variable “Zone”), experiment treatment (variable “Experiment”), day of the vegetation, and their interrelation, *R_adj_^2^* = 0.66, *F*-ratio = 20.9, *p* < 0.001, regression coefficient on the continual predictor “Day” (vegetation duration) is 0.56±0.21

| Predictor | SS | Degr. of | MS | *F*-ratio | *p*-value |
| --- | --- | --- | --- | --- | --- |
| Intercept | 2.3 | 1 | 2.3 | 5.8 | 0.01 |
| Zone | 0.8 | 2 | 0.4 | 1.0 | 0.377 |
| Experiment | 41.3 | 4 | 10.3 | 26.3 | 0.000 |
| Day | 2.9 | 1 | 2.9 | 7.4 | 0.007 |
| Zone*Experiment | 2.0 | 8 | 0.2 | 0.6 | 0.747 |
| Error | 59.7 | 152 | 0.4 | 0.0 | 0.000 |

Notes: Zone – the effect of the plant zone (roots, leaves, stems), experiment – the effect of the experiment treatments, Day – day duration of the vegetation in time of the data collection, Zone*Experiment, Zone*Day, Experiment*Day – the interrelation effects of the corresponding ordinal factors, SS – sum of squares, MS – mean of the sum of squares
